# Identification of a Rare Branch Point Variant in the *SMS* Gene in a Large Family With a Severe Form of Snyder–Robinson Syndrome

**DOI:** 10.1111/cge.14643

**Published:** 2024-11-10

**Authors:** Antoine Civit, Nathalie Ronce, Benjamin Cogné, Thomas Besnard, David Laurenceau, Catherine Hubert, Marie‐Pierre Moizard, Paul Gueguen, Annick Toutain, Marie‐Laure Vuillaume

**Affiliations:** ^1^ Service de Génétique, Centre Hospitalier Régional Universitaire de Tours Tours France; ^2^ Nantes Université, CHU de Nantes, CNRS, INSERM, l'institut du thorax Nantes France; ^3^ Nantes Université, CHU de Nantes, Service de Génétique médicale Nantes France; ^4^ UMR 1253, iBrain, Université de Tours, INSERM Tours France

**Keywords:** branch point variant, RNA‐Seq, *SMS*, Snyder–Robinson syndrome

## Abstract

Identification of the first pathogenic branch point variant in the *SMS* gene in a large French non‐consanguineous family with a phenotype retrospectively consistent with Snyder–Robinson syndrome. RT‐PCR analysis followed by RNA‐sequencing demonstrated that this variant, lead to the synthesis of a predominant aberrant transcript with complete intron 6 retention.

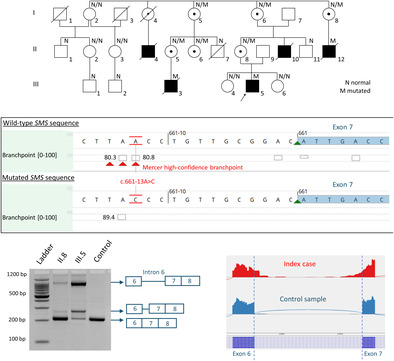

To the Editor

Snyder–Robinson syndrome (SRS) is a rare X‐linked recessive syndrome characterized by intellectual disability, speech abnormalities, epilepsy, osteoporosis, low muscle bulk, and dysmorphic features and caused by hemizygous loss‐of‐function variants in the *SMS* gene.

In 1999, our group reported a large X‐linked intellectual disability French family with hypotonia, severe early‐onset seizures, muscle hypodevelopment, bone demineralization, and dysmorphic craniofacial features, which retrospectively fits with SRS (Figure 1A) [1]. Informed consents signed by legal guardians and/or patients for genetic studies were obtained. Linkage analysis highlighted a 11.2 Mb candidate region in the Xp proximal region (Xp22.13‐Xp21.2) but subsequent X‐exome sequencing failed to identify the causative gene. Recently, we identified by exome sequencing in the proband a hemizygous branch point (BP) variant NM_004595.5(SMS):c.661‐13A>C inherited from the healthy mother, in the intron 6 of the *SMS* gene (Figure 1B). This variant, located within the linkage region, segregated with the disease in this family and was predicted to affect the BP motif relevant for physiological *SMS* pre‐mRNA splicing (Figure 1B). BP variants may compromise the assembly of the spliceosome, leading to dramatic changes in splicing and gene expression with disease consequences. BPs are difficult to identify in the human genome as their surrounding sequences show high degeneracy. Until now, only few diseases caused by BP mutations have been described and in most cases, these BP variants result in complete/partial exon skipping or intron retention or a combination of both [2].

**FIGURE 1:**
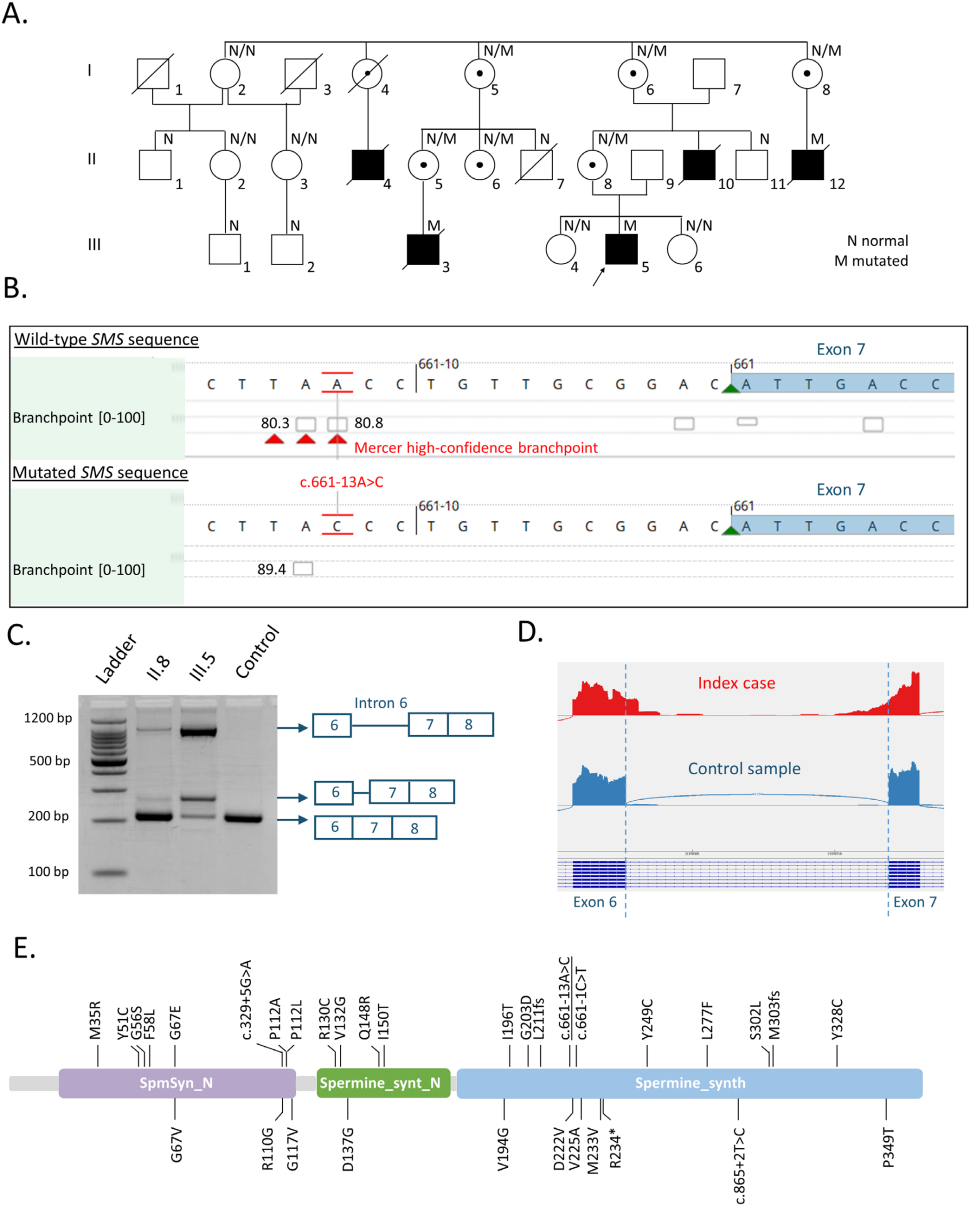
(A) Partial pedigree of the family. (B) Overview of the region encompassing the *SMS* variant (Alamut Visual Plus software). (C) RT‐PCR products using primers spanning exon 6–8 of *SMS*. (D) Sashimi plot from Integrative Genome Viewer (IGV) of exome targeted RNA‐seq data. (E) *SMS* variants reported in the literature or in ClinVar and DECIPHER databases (upper and lower panel).

RT‐PCR analysis conducted on patient's mRNA showed a predicted 205 bp fragment containing *SMS* wild‐type mRNA and two additional mRNA fragments measuring approximately 250 and 1000 bp in the proband (Figure 1C). Exome targeted RNA‐seq data only showed the presence of a predominant aberrant transcript with a complete inclusion of intron 6, probably corresponding to the longest abnormal fragment detected by RT‐PCR. Due to intron retention, this aberrant transcript could escape NMD by being temporally sequestered in the nucleus, as it was suggested in some studies focusing on transcripts with retained introns.

Until now, 32 hemizygous pathogenic variants have been described in both literature, ClinVar and DECIPHER databases including 26 different missense variants, one nonsense variant, three splice variants and two frameshift variants (Figure 1E) [3, 4, 5]. In the previously reported SRS patients, there is a wide phenotypic variability and most of the variants described so far, are hypomorphic missense variants affecting protein folding and dimerization and probably resulting in reduced spermine synthase activity. Only one hemizygous truncating variant was associated with a total absence of the protein. This variant, identified in a male patient deceased at 4 months of age because of severe multiple congenital anomalies, was suggested to lead to the most severe form of the disease [3]. By comparison, the less severe, and typical of SRS, phenotype observed in the affected individuals of our family, could be explained by the presence of a very small amount of the wild type transcript leading to a low residual activity of the protein. Unfortunately, we were not able to measure the spermine synthase activity in the patient's cell nor determine if this variant was associated with a residual presence of SMS protein.

In conclusion, we have identified a novel variant in *SMS* which disrupts a predicted BP leading to a complete retention of intron 6. To our knowledge, this is the first report of a mutation that disrupts a functional BP sequence in SRS. Our results suggest that the phenotype observed in this family is caused by aberrant splicing of the *SMS* gene. This study also brings the end to the diagnostic course of this family, allowing appropriate genetic counseling to the new generations and emphasizes the importance of focusing on noncoding regions to improve molecular diagnosis.

## Conflicts of Interest

The authors declare no conflicts of interest.

## Data Availability

Research data are not shared.
